# Evaluation of the Accuracy and Quality of Information in Videos About Lateral Epicondylitis Shared on Internet Video Sharing Services

**DOI:** 10.7759/cureus.22583

**Published:** 2022-02-24

**Authors:** Bekir Karagoz, Murat Bakir, Tolga Kececi

**Affiliations:** 1 Department of Orthopaedics and Traumatology, Adıyaman University Training and Research Hospital, Adıyaman, TUR; 2 Department of Orthopaedics and Traumatology, Health Sciences University, Sultan Abdulhamid Han Training and Research Hospital, Istanbul, TUR; 3 Department of Orthopaedics and Traumatology, Ordu University Training and Research Hospital, Ordu, TUR

**Keywords:** quality, video, patient education, youtube, lateral epicondylitis

## Abstract

Purpose

In this study, it was aimed to determine the quality and accuracy of the videos on YouTube about lateral epicondylitis.

Methods

The first 100 videos were included in the study by typing the keyword "lateral epicondylitis" in the YouTube search tab without using any filters. The video power index (VPI) was used to evaluate the popularity of the videos, and the global quality score (GQS), Journal of the American Medical Association (JAMA), and DISCERN scoring systems were used to evaluate the quality. The obtained data were statistically analyzed according to these scoring systems.

Results

The mean DISCERN, JAMA, and GQS of the analyzed videos were 46.66, 3.13, and 3.85, respectively. According to these results, it was determined that the videos were of medium quality. A statistically insignificant and weak correlation was found between the VPI and DISCERN, GQS, and JAMA scores (p>0.05, intraclass correlation coefficient, ICC: −0.05, 0.09, and −0.05, respectively). While there was no significant relationship between the video source and the DISCERN, JAMA, and GQS scores (p>0.05), it was determined that the DISCERN, JAMA, and GQS scores in the exercise videos were significantly higher than in the other content types in terms of the video content (p=0.041).

Conclusions

According to the results obtained, it was determined that YouTube videos about lateral epicondylitis were not of sufficient quality. In order to ensure standardization for quality videos, internationally acceptable guidelines should be determined and studies should be carried out to provide an adequate infrastructure for the preparation of quality medical videos that can meet the increasing needs of patients by health institutions.

## Introduction

The Internet, as a source of health care information, has become increasingly important over the years. It has been shown that 80% of the adult population in the United States has researched health issues on the internet at least once [1]. YouTube, a video platform, is one of the most popular websites, with 300 hours of video uploaded per minute and more than one billion views every month [2-4].

Uploading videos to the YouTube platform is simple and free. This situation causes controversy about video quality and reliability. Patients can use YouTube to access health-related information they are curious about. However, uploaded videos may lead to insufficient, misleading, or misinformation due to insufficient peer review [5].

Recently, concerns have been raised about the accuracy of health-related videos available on YouTube [2,6]. Doctors need to be aware of the quality of the content, as information on YouTube can affect the patient-clinician relationship and patient decision-making [7]. However, given the prevalence of internet searches and the unregulated quality of medical information provided, physicians should help their patients be adequately informed [8].

Lateral epicondylitis is common tendinopathy that can cause pain and thus loss of productivity [9]. It is known as tennis elbow because it affects 50% of tennis players, especially beginners who are learning a one-handed backhand [10]. However, lateral epicondylitis is a serious health problem due to its high incidence in manual workers [11]. Lateral epicondylitis usually resolves spontaneously during follow-up without treatment [12]. There are many videos about lateral epicondylitis on YouTube. However, in the literature review, there is no study evaluating the quality and accuracy of these videos. In this study, it was aimed to determine the quality and accuracy of the information on YouTube about lateral epicondylitis.

## Materials and methods

A search was conducted using the keywords "lateral epicondylitis" on YouTube (http://www.youtube.com) on August 20th, 2021, in Adiyaman, Turkey. The search was conducted in a web browser with no recorded history and no "cookies," without any changes to the YouTube search options or any filters. Studies of user behaviors in internet search engines have shown that more than 90% of users are interested in the results on the first three pages of search results [13]. For this reason, in this study, the search results were limited to the first 100 videos that did not have exclusion criteria. The exclusion criteria were (1) silent videos, (2) non-English videos, (3) videos less than one minute in duration, (4) commercial videos, (5) videos with less than 10,000 views, and (6) repetitive videos. To evaluate the popularity of the first 100 videos included in the study, the number of views, time elapsed since the upload date, rate of views, number of comments, and number of likes and dislikes were recorded. Apart from this, the video length (seconds), video content, and video source were recorded. Video resources were classified as doctors, associate professionals (healthcare workers other than licensed medical practitioners, such as physiotherapists or alternative medicine providers), patients, private clinics, and commercial. The videos evaluated were divided into five categories in terms of content: (1) exercise training, (2) disease presentation, (3) patient experiences, (4) surgical technique, and (5) non-surgical treatment videos.

The video power index (VPI), which was used to evaluate video view interaction and to avoid the YouTube ranking algorithm parameters due to commercial concerns, was calculated using the formula (like rate × view rate/100) [14]. While calculating the like rate, the formula (like rate * 100/(like + dislike rate)) was used, and the formula (views/time (days)) was used when calculating the view rate. The DISCERN, global quality score (GQS), and Journal of the American Medical Association (JAMA) scoring systems were used to evaluate the video quality and accuracy. These scoring systems were evaluated independently by two senior orthopedic and traumatology surgeons. Disputes regarding video reviews were resolved by discussion and consensus. DISCERN was created to assess the quality of the information provided by the University of Oxford (Table 1) [15]. The scoring system consists of 16 questions in total. Each question is scored from 1 to 5, resulting in a total score of 16 to 80. Results are considered the excellent quality between 63 and 75 points, the good quality between 51 and 62 points, the medium quality between 39 and 50 points, the poor quality between 27 and 38 points, and the very poor quality between 16 and 26 points. The JAMA scoring system is used to evaluate video accuracy and reliability (Table 2) [16]. It consists of four criteria (authority, quality, clarity, and currency) and each criterion is scored between one and four points. One point indicates low level, two and three points indicate partially medium level, and four points indicate high source accuracy. The GQS scoring system is used to evaluate the educational value of videos. It consists of five questions, and each question is valued at one point (Table 3) [17]. One point is considered low quality, and five points is excellent quality. No human subjects were included in the current study. Therefore, it was exempted from institutional review by our ethics committee as it only involved the use of public access data.

**Table 1:** DISCERN scoring system.

Question number	What Is investigated?	Question rating				
		No partially, Yes				
Section 1	Are the aims clear?	1	2	3	4	5
	Does it achieve its aims?	1	2	3	4	5
	Is it relevant?	1	2	3	4	5
	Is it clear what sources of information were used to compile the publication (other than the author or producer)?	1	2	3	4	5
	Is it clear when the information used or reported in the publication was produced?	1	2	3	4	5
	Is it balanced and unbiased?	1	2	3	4	5
	Does it provide details of additional sources of support and information?	1	2	3	4	5
	Does it refer to areas of uncertainty?	1	2	3	4	5
Section 2	Does it describe how each treatment works?	1	2	3	4	5
	Does it describe the benefits of each treatment?	1	2	3	4	5
	Does it describe the risks of each treatment?	1	2	3	4	5
	Does it describe what would happen if no treatment is used?	1	2	3	4	5
	Does it describe how the treatment choices affect overall quality of life?	1	2	3	4	5
	Is it clear that there may be more than one possible treatment choice?	1	2	3	4	5
	Does it provide support for shared decision making?	1	2	3	4	5
Section 3	Based on the answers to all of these questions, rate the overall quality of the publication as a source of information about treatment choices	1	2	3	4	5

**Table 2:** The Journal of American Medical Association (JAMA) criteria.

Criteria	Description
Authorship	Authors and contributors, their affiliations, and relevant credentials should be provided
Attribution	References and sources for all content should be listed clearly, and all relevant copyright information noted
Disclosure	Web site "ownership" should be prominently and fully disclosed, as should any sponsorship, advertising, underwriting, commercial funding
Currency	Dates that content was posted and updated should be indicated

**Table 3:** Global quality score (GQS).

1	Poor quality; very unlikely to be of any use to patients
2	Poor quality but some information present; of very limited use to patients
3	Suboptimal flow, some information covered but important topics missing; somewhat useful to patients
4	Good quality and flow, most important topics covered; useful to patients
5	Excellent quality and flow; highly useful to patients

Statistical analysis

SPSS 23.0 (IBM Corp., NY, USA) was used as the statistical analysis program. Statistically analyzed were: the VPI using the DISCERN, JAMA, and GQS scores; video source using the VPI, DISCERN, JAMA, and GQS scores; video content using the DISCERN, JAMA, and GQS scores; view rate using the VPI and DISCERN scores; and the relationships between the JAMA scores and view rate and video source. Normally distributed quantitative variables were compared with the independent group t test. A Spearman correlation analysis was used to evaluate the relationships between quantitative variables. Interobserver reliability for each score was determined by calculating the intraclass correlation coefficient (ICC) [18].

## Results

In the current study, 100 videos were evaluated. Descriptive statistics are given in Table 4. According to the DISCERN scoring, 16 (16%) videos were of excellent quality, 14 (14%) were of good quality, 45 (45%) were of medium quality, 23 (23%) were of poor quality, and two (2%) were of very poor quality. It was determined that the JAMA score was low, with one point in 11 (11%) videos, moderate with two and three points in 44 (44%) videos, and high with four points in 45 (45%) videos. The GQS score was calculated as one point for two (2%) videos, which were shown to be of poor quality, while 39 (39%) videos were recorded as excellent quality, with five points.

**Table 4:** Descriptive statistics. VPI: video power index, JAMA: Journal of American Medical Association, GQS: global quality score.

	Minimum value	Maximum value	Mean	Standard deviation
Video duration (seconds)	63	1,235	387.24	24.81
Views	10,400	2,247,404	19,398.126	396.59
Likes	0	27,000	1770	46.69
Dislikes	0	1,700	77.14	21.2
Comments	0	1,757	125.59	26.7
Like ratio	3,555	9,961	92.55	8.34
View ratio	407	141,666	114.83	26.42
VPI	283	135,293	108.94	21.84
DISCERN	16	75	46.66	13.45
JAMA	1	5	3.13	1.01
GQS	1	5	3.85	1.14

Analysis to evaluate the relationship of VPI with DISCERN, GQS, and JAMA scores found a statistically insignificant and weak correlation (p>0.05, ICC=−0.05, 0.09, and −0.05, respectively). JAMA results were moderately correlated with DISCERN and GQS results (p<0.05, ICC=0.559 and 0.541, respectively), while a very strong correlation was found in correlating DISCERN results with GQS results (p<0.05, ICC=0.983). Table 5 shows the mean, standard deviation, and median values of the scores of the VPI, DISCERN, GQS, and JAMA scores according to the video source and content.

**Table 5:** Mean, standard deviation, and median values of the JAMA, DISCERN, GQS, and VPI scores according to video source and content. VPI: video power index; JAMA: Journal of American Medical Association; GQS: global quality score.

	JAMA mean ± SD (median)	DISCERN mean ± SD (median)	GQS mean ± SD (median)	VPI median ± SD (median)
Video sources				
Doctor	3.28 ± 0.16 (4)	49.59 ± 2.48 (45.5)	4.02 ± 0.19 (4.5)	87.24 ± 33.23 (20.98)
Patient	2	43	4	19.84
Private clinics	3 ± 0.45 (3)	44.2 ± 8.28 (42)	3.2 ± 0.73 (2)	44.64 ± 9.22 (47.40)
Allied health personnel	3.08 ± 0.14 (3)	44.73 ± 1.44 (43.5)	3.83 ± 0.14 (4)	73.73 ± 17.82 (33.07)
Commercial	2 ± 0.7 (1.5)	43.00 ± 7.08 (37)	3 ± 0.71 (2.5)	42.6 ± 23 (28.73)
Total	3.13 ± 0.1 (3)	46.66 ± 1.36 (43)	3.85 ± 0.11 (4)	58.61 ± 21.89 (47.7)
Content type				
Exercise education	3.84 ± 0.17 (3)	49.56 ± 2.02 (46)	4.32 ± 0.17 (5)	131.37 ± 55.82 (41.94)
Description of disease	3.27 ± 0.17 (3.5)	46.93 ± 2.52 (43.5)	3.93 ± 0.23 (4)	109.36 ± 53.74 (47.80)
Patient experiences	2.25 ± 0.48 (2.5)	43.75 ± 2.75 (43.5)	3 ± 0.41 (3)	32.5 ± 9.78 (37.60)
Surgical technique video	2.89 ± 0.43 (3)	45.22 ± 6.29 (42)	3.22 ± 0.45 (3)	32.50 ± 10.90 (20.11)
Non-surgical technique video	3.09 ± 0.20 (3)	47.25 ± 2.65 (42)	3.69 ± 0.19 (4)	75.91 ± 14.50 (45.52)
Total	3.13 ± 0.10 (3)	46.66 ± 1.35 (43)	3.85 ± 0.11 (4)	78.95 ± 21.89 (37.70)

There was no significant correlation between the video source and VPI, DISCERN, GQS, and JAMA scores (p>0.05). However, although there was no significant relationship, it was determined that VPI, DISCERN, GQS, and JAMA scores were higher in videos made by doctors.

According to the analysis performed to evaluate the relationship between the video content and the VPI, DISCERN, JAMA, and GQS scores, it was determined that the VPI, DISCERN, JAMA, and GQS scores of the exercise training videos were statistically significantly higher (p=0.041).

In the analysis performed to evaluate the relationship between the viewing rate and video source, no statistically significant relationship was found (p>0.05). However, since the viewing rates of the videos from the doctors and allied health personnel were high, if the videos from other sources were excluded from the evaluation due to their scarcity, there was a statistically significant difference between the viewing rates of the videos from the doctors and allied health personnel (p=0.019). The rate of viewing videos originating from assistant health personnel was significantly higher than the rate of viewing videos originating from doctors.

In the analysis made between the viewing rate and the VPI, DISCERN, GQS, and JAMA scores, there was a positive significant relationship with the VPI, while negative significant relationships were observed between the DISCERN, GQS, and JAMA scores (p<0.05).

The ICC was used to evaluate the interobserver agreement in the scoring and no statistically significant difference was found (p<0.001, ICC=0.91).

## Discussion

The most important finding of this study is that YouTube videos on lateral epicondylitis do not provide moderately sufficient information. However, another important finding of the study was that while there was no significant relationship between the video source and the DISCERN, GQS, and JAMA scores, the DISCERN, GQS, and JAMA scores for the exercise videos were significantly higher than those of the other content types in terms of the video content.

The popularity of YouTube among patients is increasing day by day since visuality is at the forefront in online searches for health and the ease of accessing information [19]. Obtaining information online can increase patient satisfaction when the right resources are used [20,21]. However, since the quality of online information is variable and erratic, this can mislead patients and destabilize the clinician-patient relationship [22]. Accordingly, Goyal et al. found that 78% of YouTube videos about carpal tunnel syndrome contained at least one statement that could cause misunderstanding [23]. However, there are many studies in the literature for the quality assessment of videos on different topics related to health information on YouTube [24-26]. The first study conducted for this purpose was the study by Keelan et al. [24]. In their study evaluating the quality of vaccine-related videos on YouTube, Keelan et al. found low-quality scores for different medical conditions. In addition, studies in the literature on hip arthritis, lumbar surgery, anterior cruciate ligament tears, and rotator cuff tears have also reported poor results [3,26]. In the current study, it was determined that there was moderate-quality information in the scoring of the quality evaluation of YouTube videos about lateral epicondylitis. The results obtained were compatible with the literature. It is believed that the videos uploaded to YouTube should undergo certain standardization to increase the quality level of the videos.

When the existing studies on video sources in the literature are examined, the most important factor in obtaining sufficient information is the video source [7]. The video source is an indicator of the quality of the video, and the videos prepared by physicians have better information quality [27,28]. On the other hand, Dincel et al., examined videos about Achilles tendon rupture on YouTube and found that although the quality scores of the videos uploaded by the doctors were higher than the other groups, even those videos did not contain sufficient quality information [5]. In the current study, it was determined that physicians had the highest denominator, with 42% as a video source. In the DISCERN, JAMA, and GQS scores used to evaluate the quality of video sources, high-quality results, including videos uploaded by doctors, could not be obtained. Videos uploaded to YouTube contain a large amount of medical terminology, which can lead to boredom by viewers and a decrease in the number of views. In addition, physicians prepare more superficial videos with these reservations, and it is believed that this situation reduces the video quality.

Studies have shown that the "attractiveness" or "readability" of videos is as effective as the content itself on viewership rates [29]. Desai et al. found that although educational videos had accurate and reliable information, they were viewed less than low-quality videos [19]. Of the videos evaluated in this study, 25% were exercise videos, 30% were disease presentation videos, 4% were patient experience videos, 9% were surgical technique videos, and 32% were non-surgical treatment videos. Although the exercise videos had statistically higher scores in the quality assessment, it was determined that the quality level of all of the videos, including the exercise videos, was moderate. According to these results, it is also important that health information videos on YouTube contain sufficient, accurate, and understandable information about the etiology, natural course and treatment alternatives, possible consequences, and complications of the disease. However, it is believed that physicians should contribute to the preparation and updating of optimal medical videos, as they know the difficulties of manipulating self-educated patients.

This study had several limitations. First, the top 100 videos found on YouTube in response to the search for "lateral epicondylitis" were evaluated. This was a limitation, but there was a study in the literature showing that internet users considered the first two pages of their results when searching for a keyword [30]. Another limitation is that when this study was designed, analysis was not performed for another term that might be common, such as "tennis elbow" or "elbow pain," rather than the clinical term "lateral epicondylitis." Finally, YouTube and the internet are growing platforms. There is a steady increase in video and content. Therefore, researchers may obtain different results at different times in their studies. This situation can be considered as one of the limitations of the study.

## Conclusions

The number of videos on YouTube where patients can get information about lateral epicondylitis is increasing day by day. In our study, although the quality scores of the videos prepared by the doctors were slightly higher, it was determined that the videos were not of sufficient quality in general. In this context, in order to increase the quality of online patient education, physicians should be able to highlight less discussed topics in videos and prepare easy-to-understand videos where they can answer patients' questions. However, to ensure standardization for higher quality videos, internationally accepted instructions should be determined, and studies should be carried out to provide sufficient infrastructure for preparing medical videos of a quality that can meet the increasing needs of patients by health institutions. In addition, more healthcare information videos refined by a better filtering process created by health care providers will increase public health awareness on a platform like YouTube, which can be a powerful tool for delivering the right information to the public.
